# Iris Versicolor

**Published:** 1889-05

**Authors:** A. McNeil


### IRIS VERSICOLOR.

This drug has been lauded as a specific for sick headache. Beware of this and all such delusions. There are no specifics for diseases, but every drug is a specific for a certain group of symptoms, and I will endeavor to show those which are curable by Iris.

It is indicated in sick headache which begins with a blur before the eyes. Kali bichrom. cures blindness, followed by violent headache; the vision returns as the headache increases. Gelsemium also has headache preceded by blindness. It cures another form of headache, in which there is dull throbbing or shooting in the right side of forehead, attended by nausea, is worse toward evening, from rest, cold air, and coughing, and is ameliorated by moderate motion. Ferrum aceticum is somewhat like it, being ameliorated by slight motion; while Sepia is directly contrary to it, being aggravated by moderate motion and relieved by continuous hard exercise in its headaches and other forms and complaints, as, for instance, asthma relieved by dancing is cured by it. Iris has a headache of sharp, cutting pains of short duration, and changing location often.

Iris is to be thought of when the mouth and tongue feel as though they had been scalded; Apis and Sepia also; while, with Sanguinaria the tongue alone feels as if scalded. Iris, with many other remedies, has salivation, but it has a symptom accompanying which differentiates it from that of all other drugs—viz.: the gums and tongue feel as if covered with a greasy substance. This peculiar feeling should be borne in mind in gastric conditions, including sick headaches.

Iris is indicated in any of the diseases of the throat, including diphtheria, when it smarts and burns, with a feeling of enlargement, as if it were a burning cavern.

We should remember this remedy when milk disagrees, it becomes sour, and is thrown up. In Æthusa, the milk comes up in clots, and the vomiting of milk, which is characteristic of Mercurius is, like that of Iris, sour.

Iris has a large field of usefulness in gastric derangements. It is useful in nausea and vomiting of sour food (Calcarea carb. and Chamom.), the whole person smells sour. Hyperic., Magnesia carb., Rheum, and Sulphuric acid all have the sour smell of the person. It is indicated in vomiting of thin, watery fluid of an exceedingly sour taste.

It is the remedy in a peculiar vomiting of an extremely sour fluid which excoriates the throat, with a burning in the mouth down into the stomach.

Iris is curative in diarrhœa of watery stools, the anus feels on fire: this burning may be either at the anus, or it may extend through the whole alimentary canal, from the mouth. Arsenicum is also characterized by this burning at the anus, but the other symptoms of these drugs are too different to embarrass you in your selection.

A. McNeil.

